# Tertiary lymphoid structures in the tumor immune microenvironment of EGFR mutation–positive lung cancer are associated with prognosis: a retrospective review

**DOI:** 10.1186/s12885-026-16644-5

**Published:** 2026-08-01

**Authors:** Hikaru Yamaguchi, Masaru Hisa, Akio Enta, Yoshiyuki Maruya, Kosuke Fujisima, Sho Inomata, Hayato Mine, Hironori Takagi, Yuki Ozaki, Masayuki Watanabe, Takuya Inoue, Takumi Yamaura, Mitsuro Fukuhara, Satoshi Muto, Naoyuki Okabe, Takeo Hasegawa, Kazuyuki Hamada, Jun Osugi, Mika Hoshino, Mitsunori Higuchi, Yutaka Shio, Hiroyuki Suzuki

**Affiliations:** Department of Chest Surgery, Fukushima Medical University, Fukushima, Japan

**Keywords:** Non-small cell lung cancer, EGFR, Tertiary lymphoid structures, Tumor microenvironment, Imaging mass cytometry, Granzyme B

## Abstract

**Background:**

The tumor immune microenvironment (TME) of epidermal growth factor receptor (EGFR)-positive non-small cell lung cancer (NSCLC) remains poorly characterized. We hypothesized that a higher abundance of tertiary lymphoid structures (TLSs) is associated with improved disease-free survival (DFS) and that TLS abundance differs across major EGFR mutation subtypes.

**Methods:**

We retrospectively analyzed the medical records of 213 patients with EGFR mutation-positive NSCLC who underwent surgical resection between 2007 and 2015. TLS was defined as the presence of lymphocyte aggregates containing peripheral nodal addressin-positive endothelial venules. Clinicopathological factors, prognosis, and spatial immune profiles were assessed using imaging mass cytometry.

**Results:**

Tumors with high TLS abundance were more frequent in the L858R than in Exon19del cases (57% vs. 37%, *P* = 0.011). In stage II–III disease, high TLS abundance remained prognostic (*P* = 0.003), particularly in L858R tumors. Univariate analysis revealed that TLS abundance is an independent prognostic factor. Imaging mass cytometry showed a higher density of granzyme B-positive cytotoxic lymphocytes in tumors with high TLS abundance.

**Conclusions:**

Higher TLS abundance was associated with improved DFS in EGFR mutation-positive NSCLC, especially in L858R tumors. TLS abundance may reflect a more active cytotoxic immune niche and contribute to differential therapeutic responses; however, mechanistic interpretations require further validation.

## Background

Lung cancer is a leading cause of cancer-related mortality worldwide, with non-small cell lung cancer (NSCLC) being the most common [1]. Multiple oncogenic driver gene mutations occur in NSCLC, among which epidermal growth factor receptor (EGFR) mutations are particularly prevalent, occurring in 20–30% of cases in Europe and the United States and in approximately 50% of cases in Asia [2].

Compared with conventional chemotherapy, molecularly targeted therapy with EGFR tyrosine kinase inhibitors (EGFR-TKIs) improves clinical outcomes in patients with EGFR mutation-positive lung cancer [3]. Recently, immune checkpoint inhibitors (ICI) have been shown to prolong the survival of patients with lung cancer. However, reports have suggested limited clinical benefits in patients with EGFR mutation-positive lung cancer [4, 5]. In contrast, TKI therapy affects the tumor microenvironment (TME) and activates the immune system [6]. Changes in tumor microimmunity occur even in EGFR mutation-positive lung cancer, suggesting that some patients may benefit from ICIs and that identifying predictive factors is desirable.

Tertiary lymphoid structures (TLSs) are ectopic lymphoid structures found in inflammatory, infected, and tumor tissues, and are composed of mature dendritic cells, T cells, and B cells forming germinal centers, as well as high endothelial venules [7, 8]. TLS in the TME is associated with a favorable prognosis in NSCLC, and our institution has reported that TLS abundance correlates with disease-free survival (DFS) in patients with stage II or higher lung cancer who undergo surgery [9–11]. However, the relationship between the TLS and the prognosis of EGFR-positive lung cancer remains unclear.

Thus, in this study, we hypothesized that TLS may improve DFS even in patients with EGFR mutation-positive NSCLC and that TLS abundance might differ between Exon19del-positive and L858R-positive tumors.

## Materials and methods

### Patient selection

We retrospectively reviewed medical records of consecutive NSCLC patients with confirmed EGFR Exon19del or L858R mutations using the Cobas EGFR mutation test, who underwent initial surgical resection or diagnostic tumor biopsy at Fukushima Medical University Hospital between 2007 and 2015. Patients who had received preoperative chemotherapy or radiotherapy were excluded from the study. All patients were restaged according to the 8th edition TNM classification. As this was a retrospective study that included all consecutive eligible cases during the study period, no a priori sample size calculations were performed. Patients with uncommon EGFR mutations, including exon 20 insertions, were excluded.

### Ethics statement

This study was approved by the Ethics Committee of the Fukushima Medical University (approval no. REC2024-136). This study was conducted in accordance with the Declaration of Helsinki, and written informed consent was obtained from all participants.

### Evaluation of TLS

**Fig. 1:**
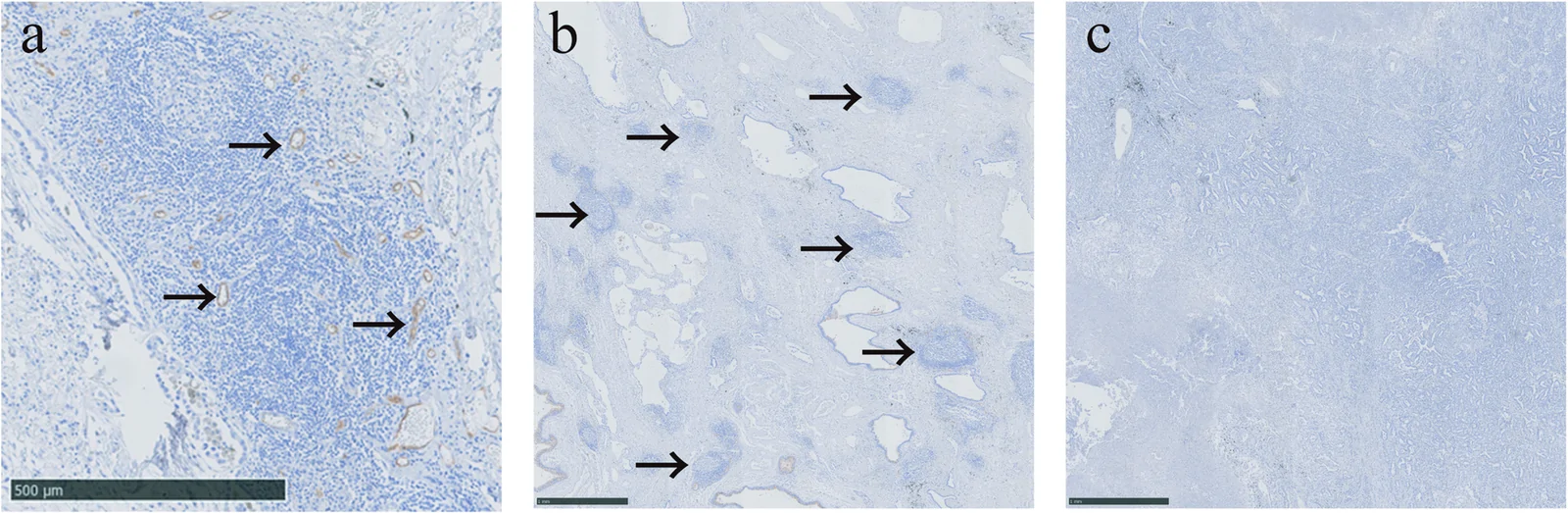
TLS classification. **a **PNAd immunostaining (brown) identifying endothelial venules (arrowheads) at ×40 magnification. **b** Representative TLS-high cases showing dense lymphoid aggregates with multiple PNAd-positive HEVs (arrows). **c** Representative TLS-low case showing sparse lymphoid clusters with few or no HEVs

TLS was defined as a PNAd-positive lymphocyte cluster containing the hepatitis E virus. PNAd immunostaining was performed under the same conditions. The number of TLS was assessed across 10 low-magnification fields. TLS density was graded as follows: −, None; +, 1–5 TLS; ++, 6–10 TLS; and +++, 10 or more TLS. TLS-high was defined as + + or +++, and TLS-low was defined as − or + (Fig. 1c).

Two blinded observers independently assessed the TLS abundance, and inter-observer agreement was high (κ = 0.78).

### Statistical analysis

DFS was defined as the interval between surgery and radiologically confirmed disease recurrence or death. Patients with stage IV disease (*n* = 7) were excluded from the DFS analysis. A total of 206 patients (stages I–III) were included in the Cox regression analysis. Kaplan–Meier curves were compared using log-rank tests. Univariate and multivariate analyses were conducted using the Cox proportional hazards regression. Variables with *P* < 0.05 in the univariate analysis were included in the multivariate models. Statistical significance was set at *P* < 0.05. Baseline factors included TLS values, age, sex, smoking history, pathological stage, EGFR mutation, and programmed death ligand 1 (PD-L1) expression. All statistical analyses were performed using SPSS version 29 software (IBM, Armonk, NY, USA).

### Panel and antibody conjugation

This study used the Human Immuno Oncology IMC™ Panel (201509), comprising 31 antibodies, which enables the differentiation of diverse immune cell types, intercellular components, and tumor cells within the TME. These antibodies were conjugated to metal tags to label both cell surface and intracellular proteins.

### Imaging mass cytometry (IMC, Hyperion XTi image scanning)

IMC was performed in 4 representative cases (TLS-high, *n* = 2; TLS-low, *n* = 2). Ten regions of interest per case were selected based on histological representativeness. Quantification was performed at the patient level using mixed-effects models to account for intra-patient correlations.

The scanning module uses a high-precision laser ablation system to scan each point of the marked tissue section samples. The maximum square area scanned was 1000 × 1000 μm, and the laser beam cross-section was 1 × 1 μm. The images were analyzed using a Hyperion XTi imaging system (Fluidigm Corp., Boston, MA, USA).

### Date procedure and normalization

The original scan files (MCD files) were exported as individual TIFF files for each antibody using the MCD viewer software. Quantification of each antibody at the cellular level was performed using the Visiopharm software.

## Results

### Patient characteristics

**Fig. 2:**
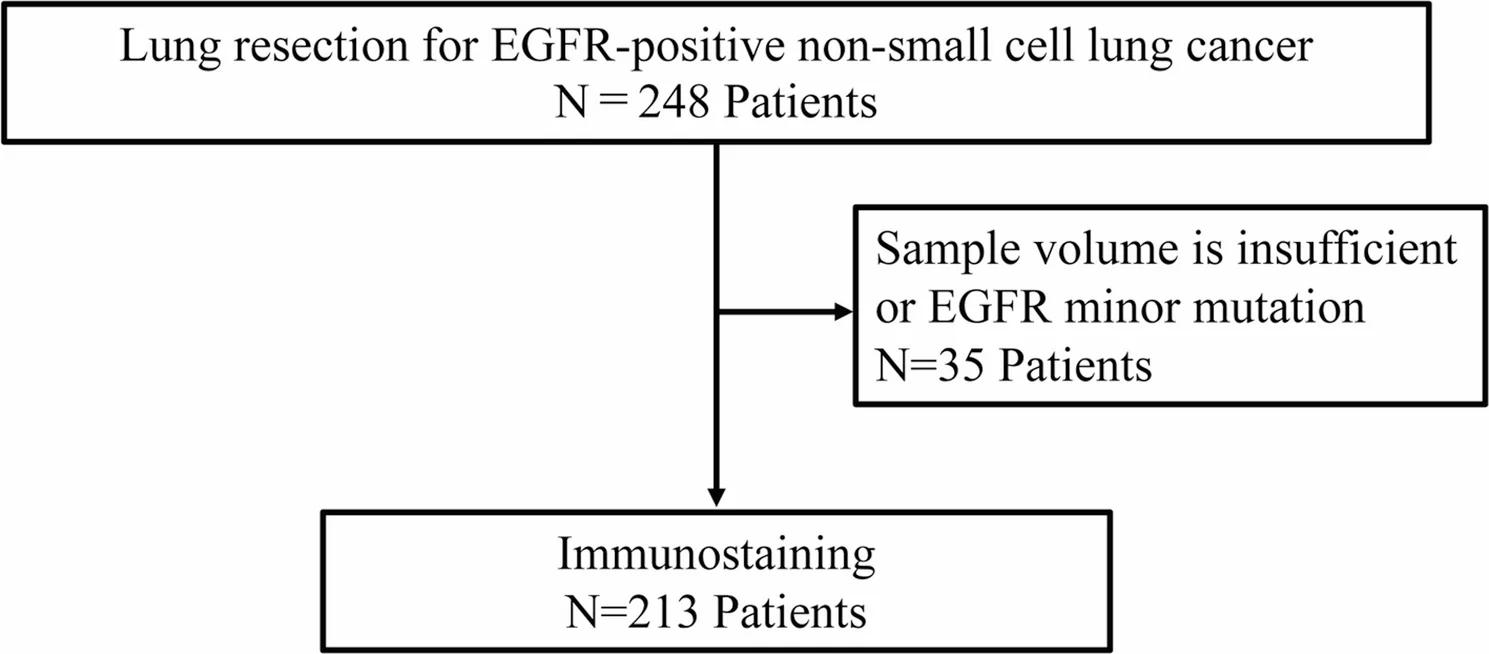
Flowchart of the patient selection process

Between January 2007 and December 2015, 248 patients were identified; 213 met the eligibility criteria and were included in the analysis (Fig. 2). The demographic and clinicopathological characteristics are shown in Table 1. The age at surgery or biopsy ranged from 37 to 90 years (median: 67 years). The cohort included 141 female patients (66.2%), 151 nonsmokers (70.8%), 209 patients with adenocarcinomas (99.2%), and 172 patients with pathological stage I tumors (80.8%) (Table 1).

**Table 1:** Clinicopathologic characteristics of the patients and TLS

Characteristics (*n* = 213)		TLS		*P*
	Overall	High	Low	
Age (y.o.)				0.94
<67	97	48	49	
≧67	106	58	58	
Sex				0.36
Male	72	39	33	
Female	141	67	74	
Smoking status				0.62
Never	150	73	77	
Former or Current	63	33	30	
Histology				0.361
Adenocarcinoma	209	103	106	
Adenosquamous cell carcinoma	2	0	2	
Squamous cell carcinoma	2	1	1	
p-stage				0.28
Ⅰ	172	86	86	
Ⅱ	13	7	6	
Ⅲ	21	11	9	
Ⅳ	7	1	6	
Recurrence				0.035
Yes	47	17	30	
No	166	89	77	
EGFR mutation				0.009
Exon19del	81	31	50	
L858R	132	75	57	
PD-L1				0.576
<1%	139	69	70	
1–49%	37	21	16	
>50%	18	9	9	
unknown	19	7	12	

*HR* hazard ratio, *CI* confidence interval, *EGFR* epidermal growth factor receptor, *TLS* tertiary lymphoid structures, *PD-L1* programmed death ligand-1

The TLS abundance was low in 107 patients and high in 106 patients (Table 1A). High TLS abundance was observed in 31 (37%) patients with Exon19del and 75 (57%) patients with L858R mutation, with the latter exhibiting a higher prevalence of elevated TLS abundance (*P* = 0.009). In addition, a tendency toward lower TLS abundance was observed in patients who experienced recurrence (*P* = 0.035).

Progression or relapse was observed in 47 patients who received EGFR-TKI therapy. Overall, 42 patients were treated with gefitinib, 3 with erlotinib, and 2 with afatinib.

### TLS abundance and survival

**Fig. 3:**
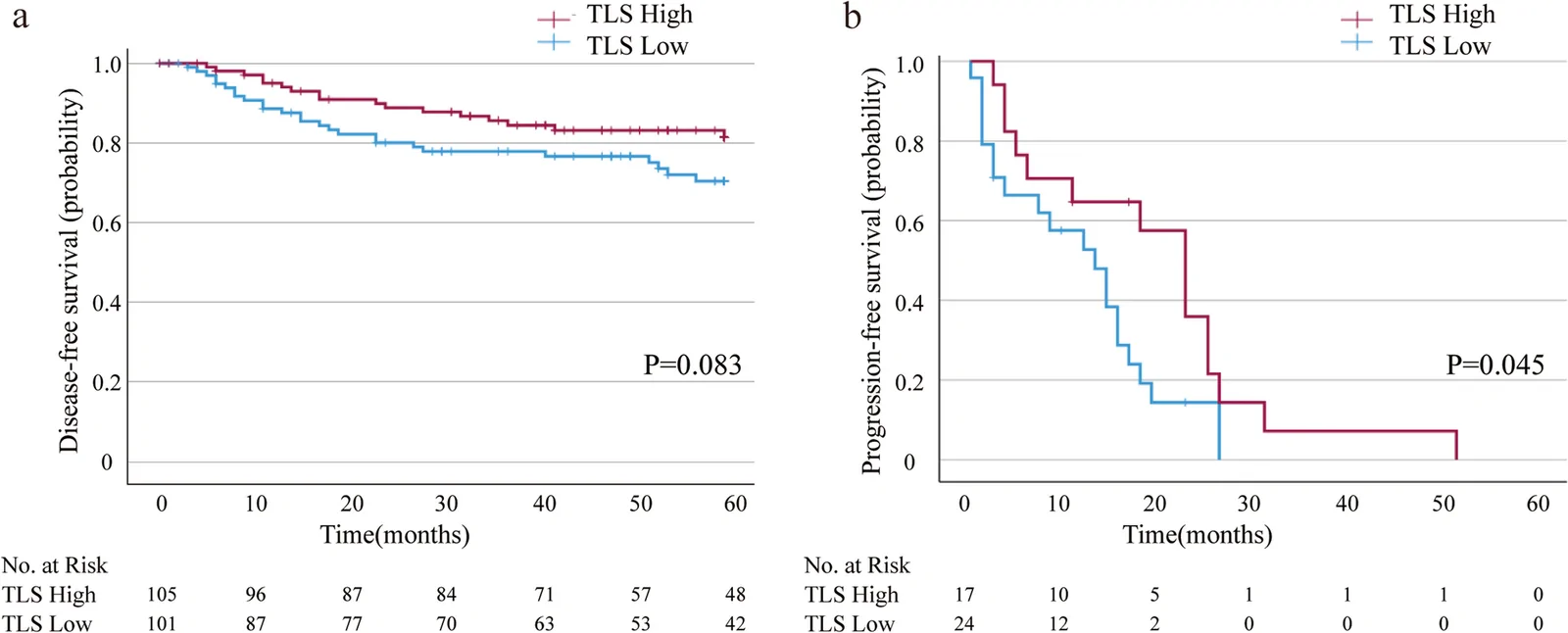
**a** Disease-free survival by TLS in StageI-III. **b** Progression-free survival in patients receiving TKI therapy after recurrence

**Fig. 4:**
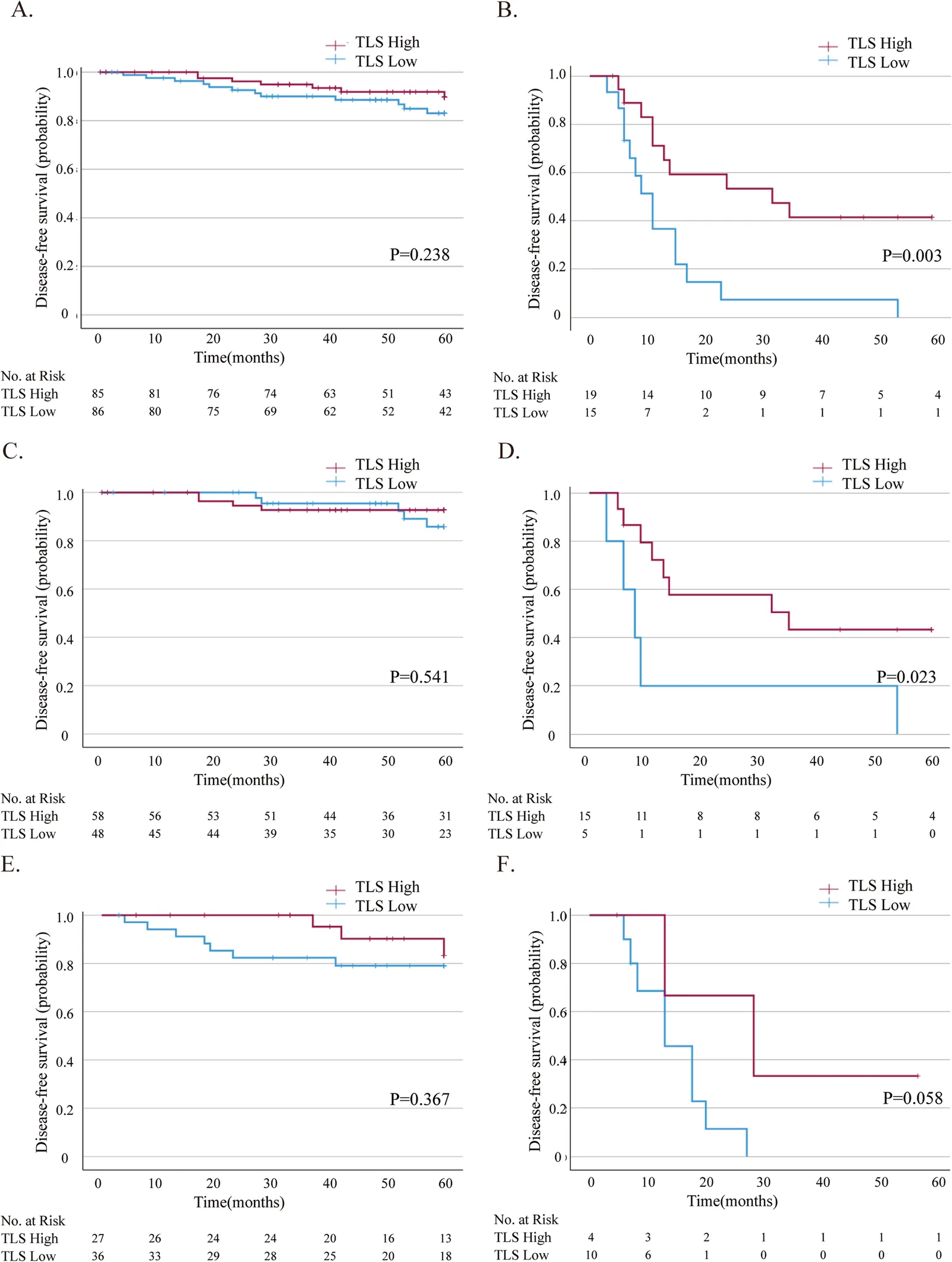
**a **Disease-free survival by TLS in patients with Stage Ⅰ disease. **b **Disease-free survival by TLS in patients with Stage Ⅱ-III disease. **c **Disease-free survival by TLS in Stage I L858R-positive cases. **d** Disease-free survival by TLS in Stage Ⅱ-III L858R-positive cases. **e **Disease-free survival by TLS in Stage I Exon19del-positive cases. **f **Disease-free survival by TLS in Stage Ⅱ-III Exon19del-positive cases

In Stage I–III cases, a lower recurrence rate was observed in the TLS-high group, but no statistically significant difference was found (*P* = 0.083) (Fig. 3a). Although 83.4% of these patients had stage I disease, the difference was not significant (*P* = 0.238) (Fig. 4a). Among stage II-III cases, patients with high TLS abundance showed significantly lower recurrence rates (*P* = 0.003) (Fig. 4b). In the L858R group, patients with high TLS abundance with stage II-III disease showed a significantly lower recurrence rate (*P* = 0.023) (Fig. 4d). No significant difference was observed in the exon 19 del cases (*P* = 0.058) (Fig. 4f). Among the 41 patients for whom follow-up data were available, progression-free survival (PFS) following recurrence after EGFR-TKI therapy was significantly better in the TLS-high group (*P* = 0.045) (Fig. 3b).

The results of the univariate analyses are presented in Table 2. Univariate analyses showed that the pathological stage classification and TLS were significant independent prognostic factors (*P* = 0.025).

**Table 2:** Univariate analyses in stage Ⅰ, Ⅱ, Ⅲ cases

Characteristics	Univariate		
	HR	95%CI	*P*
Age (years)	0.968	0.894–1.049	0.968
Sex (Male)	0.618	0.132–2.885	0.54
Smoking status (Former or Current)	0.778	0.151–4.001	0.764
p-Stage	2.5	0.523–11.956	0.251
EGFR mutation (Exon19del)	1.974	0.410–9.518	0.397
TLS (High)	0.079	0.009–0.730	0.025
PD-L1 (< 1%)	0.583	0.124–2.751	0.496

*HR* hazard ratio, *CI* confidence interval, *EGFR* epidermal growth factor receptor, *TLS* tertiary lymphoid structures, *PD-L1* programmed death ligand-1

### TLS and the TME

**Fig. 5:**
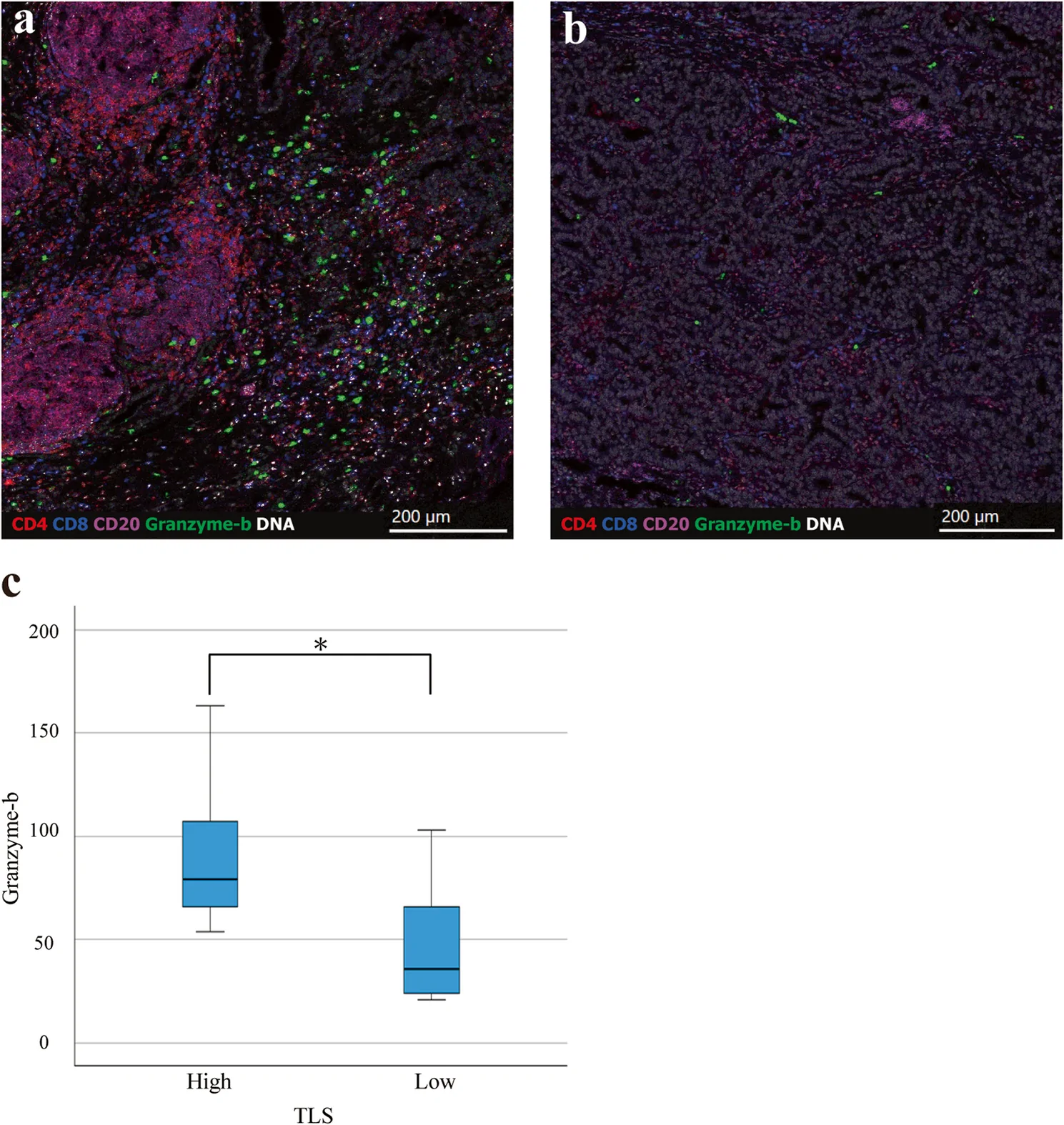
**a **IMC composite image of a TLS-high tumor. CD8+ T cells (red), CD4+ T cells (green), dendritic cells (cyan), and granzyme B (yellow) are shown. Dense cytotoxic lymphocyte infiltration is observed around TLS-rich regions. **b **IMC composite image of a TLS-low tumor showing sparse cytotoxic lymphocyte distribution. **c **Quantification of granzyme B-positive cells across 10 fields per case (TLS-high vs. TLS-low). TLS-high tumors showed significantly higher granzyme B expression (*P*=0.009)

The TMEs of the TLS-high and TLS-low cases were compared using spatial omics analysis and IMC. Differences in the localization of immune cells were confirmed (Fig. 5). Distinct spatial localization of CD8 + T cells was observed around the TLS-rich regions. In addition, the expression levels of granzyme B, a cytolytic enzyme, were analyzed using the Visiopharm software in 10 randomly selected fields of view, followed by statistical analysis. Granzyme B was significantly more frequently observed in cases with a high TLS abundance (*P* = 0.009) (Fig. 5c).

## Discussion

To date, no studies have reported an association between TLS and DFS in patients with EGFR-positive lung cancer. Exon19del is associated with improved PFS and enhanced efficacy of TKI therapy [12]. In the ATTLAS trial, a trend toward higher efficacy of ICI therapy was observed in patients with the L858R mutation compared to those with Exon19del [13].

Zhang et al. reported that the treatment efficacy of ICIs following EGFR-TKI therapy was significantly better in L858R patients than in Exon19del patients [14]. Considering these reports and the significantly higher abundance of TLSs observed in patients with the L858R mutation in our study, TLS may have influenced the therapeutic efficacy of TKIs and ICIs.

In our study, TKI treatment outcomes were significantly better in patients with high TLS. TKI therapy affects the TME, prompting transition to a hot tumor [15]. TKIs enhance major histocompatibility complex class I and II antigen presentation in response to interferon-γ, increase CD8 + T cells and dendritic cells, eliminate FOXP3 + Tregs, inhibit macrophage M2 polarization, and reduce PD-L1 expression in cancer cells [6]. In the TLS-high group, these reactions may have been more pronounced, potentially explaining the differences in TKI treatment efficacy.

The therapeutic efficacy of ICIs following TKI treatment has been reported to yield a better prognosis than other treatments [16]. IMC (Hyperion XTi) revealed that TLS-high cases exhibited significantly higher granzyme B expression and distinct immune cell localization than TLS-low cases.

Granzyme B is a serine protease produced by cytotoxic lymphocytes that reflects antitumor immune responses in tumor-infiltrating lymphocytes (TILs) [17, 18]. Kinoshita et al. reported that, in lung adenocarcinoma, a low number of granzyme B-positive TILs and low PD-L1 expression were significantly associated with poor prognosis [19]. Regarding the relationship between TIL and TLS in lung cancer, another report indicated that patients with TLS abundance showed less variation in TIL expression profiles and higher expression of lymphocytes, dendritic cells, and antigen-presenting cells [20]. TLS has been suggested to be associated with immune cell expression, particularly in guiding cytotoxic lymphocytes containing granzyme B, and exerting a dominant effect on antitumor immunity. TLS-high tumors showed higher granzyme B abundance, suggesting more active cytotoxic lymphocyte infiltration, although this did not directly indicate a fully inflamed (“hot”) tumor phenotype. Therefore, mechanistic interpretations of TKI or ICI responsiveness should be made with caution.

This study has certain limitations. The number of included cases was small, and the analysis was conducted retrospectively at a single institution, warranting caution in interpreting the generalizability of the findings. The number of specimens evaluated by IMC using the Hyperion XTi system was limited, and further validation in a larger cohort is warranted. Consequently, it was not possible to assess TLS maturity in all cases. Therefore, further prospective studies are warranted.

## Conclusions

High TLS abundance is associated with a favorable prognosis in patients with EGFR mutation-positive NSCLC, especially L858R tumors. TLS abundance may reflect cytotoxic immune activity within the TME; however, its predictive value for TKI or ICI therapy requires further investigation.

## Data Availability

The clinical datasets and imaging mass cytometry data generated and analyzed in this study contain patient‑identifiable information and cannot be publicly shared due to ethical and privacy restrictions. De‑identified data may be made available from the corresponding author upon reasonable request and with appropriate institutional approvals.

## Abbreviations

DFS: Disease-free survival
EGFR: Epidermal growth factor receptor
ICI: Immune checkpoint inhibitors
NSCLC: Non-small cell lung cancer.
PFS: Progression-free survival
TLSs: Tertiary lymphoid structures
TKIs: Tyrosine kinase inhibitors
TILs: Tumor-infiltrating lymphocytes
TME: Tumor microenvironment
